# Urological management of the patient with traumatic spinal cord injury

**Published:** 2009

**Authors:** C Persu, V Caun, I Dragomiriţeanu, P Geavlete

**Affiliations:** * Urology Department, “Sf. Ioan” Clinical Emergency Hospital – Bucharest; ** Department of Urology,“Th. Burghele” Clinical Hospital – Bucharest

**Keywords:** Spinal Cord Injury (SCI), urodynamics, neurourology

## Abstract

Traumatic spinal cord injury is a very comprehensive subject, debated in many scientific papers. It interests various medical specialties, but also other sciences, like economy, psychology or social science.

The patient having a motoric disability, with sphincter troubles and other associated pathologies secondary to a traumatic lesion of the spinal cord, represented a social problem from the antiquity. The first centers dedicated exclusively to these patients were established during Napoleon. Nevertheless, a systematic approach to these patients was not possible before the end of the Second World War, when scientific and economic development made possible the establishment of medical facilities specialized in the complex evaluation and treatment of patients with spinal cord injury (SCI).

Between the two world wars, physicians were concentrating their efforts to keep these patients alive, considering that the main target was to treat or prevent complications which could be fatal to the patient. The first scientific papers underlining the essential place of lower urinary tract disorders in the vital prognostic of the SCI patient are dating back to this time.

In modern times, the target for every patient with SCI should be social reinsertion and obtaining as much autonomy as possible. The physician needs to tune up his treatment according to this factor. The continuous evolution of medicine, alongside with technical progress and the development of modern social security have created the premises for a real quality of life of the paraplegic or even quadriplegic patient. The lower urinary tract becomes not only a key for prolonged survival, but also one of the most important elements for social reinsertion [**1**].

## Elements of neurourology

One of the essential principles of neurology states that a neurological disease can lead to the disappearance of a function or to the exacerbation of that function [**2**]. In the particular case of the lower urinary tract, loss of function means bladder paralysis and sphincter deficiency, while exacerbation of the function is represented by detrusor overactivity, usually associated with detrusor-sphincter dyssinergia, the latter being very often present in patients with traumatic SCI. The neurological classification of SCI describes three phases [**3**]:

1. Posttraumatic phase: complete abolition of contraction of all striated muscles and loss of reflex activity under the level of trauma. The bladder is areflexive, so the patient develops acute urinary retention. This period can last between two weeks and one year.

2. The recovery phase: is signaled by the recovery of reflex activity. In the urinary tract, detrusor-sphincter dyssinergia occurs, associated with detrusor overactivity (if the lesion is above the sacral spines) or with an areflexive detrusor (in sacral traumas).

3. The stable phase: it starts when no neurological changes occur.

The neurological bladder of the patient with SCI is the study model for all neurological disorders of the lower urinary tract [**4**]. The automatic activity of the bladder depends on the level of the lesion. The central neurological bladder is typical for lesions above the sacral spine, while the peripheral neurological bladder characterizes sacral traumas or lesions of the neural bundles of the urinary tract [**5**].

The central neurological bladder has a reflex activity of the detrusor, with urethral reflux and high risk for autonomic dysreflexia. The peripheral neurological bladder has no contractions, increased post void residual urine and high risk for the upper urinary tract. Detrusor-sphincter dyssinergia is present, within different stages, in all patients with central neurological bladder and in about half of the patients with peripheral neurological bladder.

## Clinical evaluation of the patient with traumatic spinal cord injury

The evaluation protocol of the patient with a neurological disorder of the bladder includes standard urological evaluation of the lower urinary tract, but also specific investigations; a very important place is occupied by the neurological examination, with all the explorations recommended by the neurologist.

The clinical examination of the SCI patient should always be carried on by several specialists due to the complexity of the pathology. The literature offers several theoretical standardizations based on the segmentation of the spinal cord, presenting the most typical findings for each level of the trauma [**6**]. These models are extremely useful from a didactic point of view, but are very often disagreed by clinical practice, which shows a huge variance of the symptoms in patients with the same level of trauma, underlining once more the importance of an interdisciplinary evaluation as the only one being able to reveal the particularities of each case.

The physician should keep in his mind that his patient has no sensitivity under the level of the trauma, thus being unable to report symptoms that would be obvious for another patient [**7**]. The perinea examination and the neurological assessment are both of high importance [**8**]. The level of each lesion and the fact that it is or it is not complete should always be mentioned in the report.

The lower urinary tract is the first one affected by the neurological disorder [**9**]. Any disorder of the upper urinary tract should be considered secondary to the deterioration of the lower tract and that’s why the first line of therapy should always aim for the bladder, one of the objectives being the health of the kidneys.

The medical history will focus on the moment of the trauma, the function of the urinary tract at that moment and the evolution of symptomatology. Any therapy will also be reported. Data concerning spasticity or episodes of autonomic dysreflexia are also very important. Any preexisting disease or abnormality will be assessed. The urologist has to be aware of the mictional choice of the patient, the presence of bladder sensations or urinary incontinence, the characters of micturition and the way of initiating it. The evaluation of the patient’s sexual activity may reveal supplemental psychogenic troubles that may additionally decrease the compliance regarding the medical therapy, or which may require specific therapy [**10**].

The bladder diary of the patient with neurogenic bladder after a traumatic lesion of the spinal cord is a particular one, being very useful in evaluating and diagnosing this patient. Considering the discrete features of each case, it will report bladder sensations, urgency, leakage associated or not with any sensations, initiation of voiding, intermittent catheterization with any specific aspects, the use of urinary condom, etc. The diary contains data regarding the intake of fluids, the volume voided and the observations of the patient. By analyzing the volume and frequency of each voiding, along with the other parameters contained in the diary, the physician will have an initial opinion about the urodynamic profile of his patient [**11**].

Urinary tract infection is present in most SCI patients [**12**] and it is due to the long period of use of the indwelling urinary catheter or to an incomplete voiding. A urine culture should be performed in all patients during each evaluation.

Abdominal ultrasound is also mandatory because it can evaluate the whole urinary tract before indicating specific, more invasive, diagnostic methods.

Uretrocystoscopy is not of high importantce in the evaluation protocol of the SCI patient, so its indications should be well argumented, considering the high risk of associated morbidity [**13**].

## The urodynamic evaluation 

The modern evaluation of the SCI patient should make use of the most modern technology available. The clinical examination cannot offer enough information, so an urodynamic evaluation is of great use for a better understanding of the lower urinary tract disorders.

Although it has limited indications in the general urological practice, the urodynamic evaluation becomes routine in patients with neurogenic bladder after a traumatic lesion of the spinal cord [**14**]. 

It is useful for the evaluation of detrusor function during filling and voiding, to assess the function of the sphincter activity and to identify the risk factors for the upper urinary tract.

Each SCI patient should undergo a complete urodynamic evaluation after the posttraumatic phase. This will become the reference for all future examinations, so a standardization of terminology and techniques is mandatory so that data obtained by different physicians can be compared [**15**]. The International Continence Society (ICS) is the authority in charge with this standardization, so that physicians around the world can speak the same language, that of the correct urodynamic evaluation.

In the posttraumatic phase the patient may not have any urological symptoms. Nevertheless, the neurogenic bladder is already there, and the alteration of the lower urinary tract has begun. The most common situation is the patient presenting spontaneous voiding but with extremely high intravesical pressure. The urodynamic evaluation reveals this situation, making it possible for an early initiation of treatment and it is also able to select cases that are more likely to develop complications. It can be used for the follow-up of the patient, indicating fine tuning or even changes in the treatment when this is no longer efficient [**16**]. The term *permanent urodynamic evaluation* becomes more and more frequent in modern literature about SCI [**17**].

The urodynamic profile will include this voiding method, data about bladder sensations and the type of neurogenic bladder. It will also contain data regarding intravesical pressure during filling and voiding or during reflex contractions. If the electromyography (EMG) diagnoses detrusor-sphincter dyssinergia, the investigator will record both bladder and urethral pressures. If the patient is symptomatic, the examination will try to reproduce these symptoms and the report should state if the symptoms were reproduced or not. If the patient is incontinent, the examination should demonstrate the incontinence and evaluate all its features [**18**]. Some authors recommend that a brief history of the spinal cord injury is also mentioned.

## Particular aspects of the urodynamic evaluation

The standard equipment for modern urodynamic evaluation should be able to perform all the standard explorations: uroflowmetry, urethral pressure profile, cystometry, EMG and pressure-flow study [**19**]. The examination should be performed in a lithotomic position, but this requires an examination table that is accessible to a disabled patient, allowing easy access and different positions of examination. ICS clearly states that bladder filing will be performed with saline, the use of gas devices being forbidden. The temperature of the saline will be around 37 degrees Celsius. The filling rate will be set at 20ml/min and a 12 Ch (8 Ch in children) catheter will be used. In most cases, the maneuvers that initiate detrusor overactivity are not necessary [**20**]. In patients at risk of autonomic dysreflexia arterial blood pressure needs to be monitorized. 

The urodynamic unit will have enough space and a layout that will allow the patient and the medical staff to circulate [**21**].

On the practical side, the examiner should be ready to cope with any particular situation he may encounter when dealing with a disabled patient – transfer from the chair to the table, continence problems, spasticity, etc. The physician should keep in mind that urinary tract infection or bladder stones represent contraindications for the urodynamic exploration and should be treated before continuing the evaluation [**22**].

The urethral pressure profile is not very useful in these patients. Moving the catheter along the urethra will trigger spasticity which will lead to erroneous results [**23**].

**Fig. 1:**
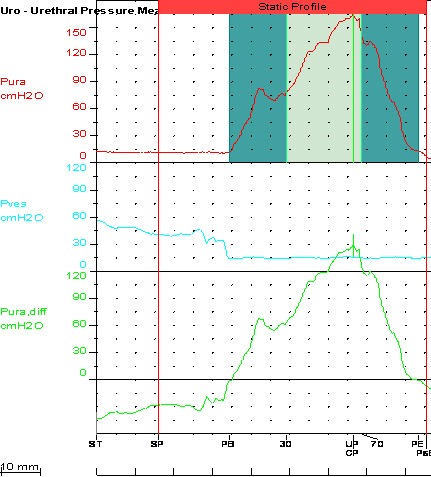
Static urethral profile

The technique for cystometry should be adapted to each patient; the signs of detrusor overactivity should be interpreted considering the filling rate is higher than the physiological one and that the catheter may irritate the bladder. Usually, the intravesical pressure is much higher than in idiopathic detrusor overactivity. The duration of each contraction is longer and it is usually associated with autonomic nervous system overactivity. The cystometry curve may look funny, being very difficult to integrate it in the clinical context of the patient [**24**]. 

**Fig. 2:**
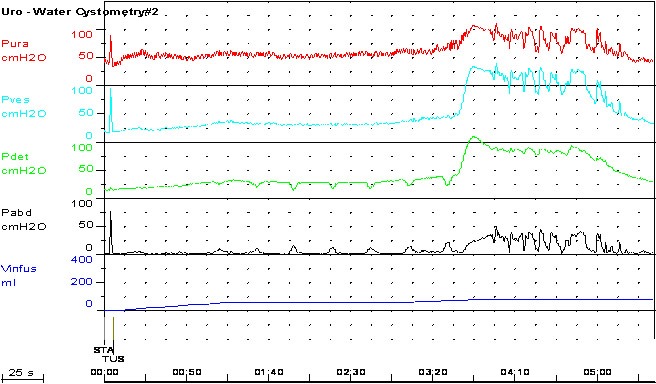
Detrusor overactivity in SCI

The pressure-flow study raises another problem to the examiner. The catheter makes obstruction which is very difficult to quantify. Some authors recommend the use of catheters smaller than 10Ch for this exploration [**25**]. The lithotomic position is also an impediment for a normal micturition so, the patient may require repositioning at the end of the filling phase and the device may need another calibration.

## The urodynamic profile of SCI patients

The voiding desire is a vast subject in this particular case. It is accepted that the desire reported by a SCI patient is different compared to healthy people. Nevertheless, the autonomous nervous system overactivity that is associated with bladder filling in these patients (sweating, arterial hypertension, etc) seems to be very specific and the patient may easily recognize the necessity to empty his bladder. The sensitivity and specificity of this type of sensation is dependent on the integrity of the autonomous nervous system. Experimental data suggests that the quality of these sensations lowers with the lowering of the level of the SCI. Amarenco introduced the term “equivalent of voiding sensation” in order to better describe the state of the patient before voiding [**26**]. A clinical trial performed on 450 patients with SCI demonstrates the presence of the equivalent sensation in 100% of the quadriplegic, in 69% of the patients with thoracic lesions and only in 29% of the patients with lower lesions [**27**].

The functional bladder capacity is determined by the occurrence of incontinence or by autonomic dysreflexia, both being bothersome factors for the patient. In patients with central neurogenic bladder, the functional bladder capacity may be as low as 30-50ml, because of the occurrence of reflex detrusor activity. If the neurogenic bladder is a peripheral type, functional bladder capacity may be very often above 1000ml [**28**].

Bladder compliance, defined as the variance of intravesical pressure during filling, is very useful for the evaluation of these patients, offering important information about the elastic properties of the detrusor. Nevertheless, because the points for measuring the compliance are arbitrary, the calculation of bladder compliance is of secondary importance. Standardization may be obtained by calculating the compliance after every 100ml infused. Compliance below 20 is considered pathological [**29**].

Detrusor contractions during cystometry are most likely pathological, being an uninhibited, reflex activity. They occur in all patients with SCI having a lesion above the sacral spine. High amplitude contractions are more frequent in higher lesions, and their frequency decreases with the lowering of the level of trauma. Bladder acontractility is associated with the destruction of the sacral medulla. 

Detrusor-sphincter dyssinergia is constant in SCI patients [**30**]. The diagnosis can only be established during cystometry with EMG. The degree of dyssinergia also seems to decrease along with the lesional level [**31**].

## Urological treatment

The main objectives of treatment are [**32**]:

• the protraction of the upper urinary tract

• complete bladder emptying

• restoration of continence

• independence for the patient

The urologists should aim at keeping the pressure of the bladder low during filling and emptying the bladder completely by intermittent catheterization or spontaneous voiding. Urinary tract infection should be early diagnosed and appropriate treatment should be started; moreover, the patient should be informed of the prophylactic measures he can take. The presence of bladder stones can be explained by the particular functionality of the detrusor, by the recurrent infections but also by an increased calcium mobilization after important traumas associated with long periods of immobility. The stone makes treatment of infection virtually impossible and may modify the features of the urodynamic profile.

A special class of patients is represented by the lesions above T6. By interrupting the nervous link between the brain and the spinal cord, autonomous activity can no longer be inhibited, creating the premises for autonomic dysreflexia, a very dangerous condition associating arterial hypertension with alteration of the cardiac activity. This state has a sudden start and may lead to death if it is not properly recognized and treated [**33**]. Most frequently, the cause of dysreflexia is urological – acute urinary retention, cystoscopy, cystometry, etc. Other causes can be extreme temperatures, sexual activity, ESWL without anesthesia, etc. The first step is to identify the cause and eliminate it. Symptomatic therapy is of limited importance if the obstruction is not eliminated, but can prove very effective as a prophylactic measure [**34**]. Some authors recommend that alpha blocker therapy should be used as prophylaxis, while others consider that endoscopic sphincterotomy is a safer option. 

There are two therapeutic options for emptying the bladder: conventional training, based on the existent reflex activity, or intermittent catheterization, a more modern choice [**35**]. These two options rely on very different urodynamic profiles, and the options for one or the other are impossible without complex urodynamic explorations.

**1. Conventional reeducation**

Conventional reeducation is targeting the voiding phase so it cannot be an option for many SCI patients [**36**]. It requires functional sacral medullar centers. If cystometry shows intravesical pressure of 40cm above water during filling, appropriate treatment is required in order to decrease the pressure. Emptying it can be triggered by increasing intravesical pressure by extrinsic maneuver or by initiating a reflex bladder contraction. In the first category, the most common are the Crédé and Valsalva maneuvers, which can be indicated in patients with acontracile or areflexive bladder [**37**]. Nevertheless, by extrinsic compression, intravesical pressure can rise dangerously, especially in patients with functional bladder outlet obstruction. Nowadays, these maneuvers are only indicated in patients having a low pressure bladder during filling and voiding, as shown on the urodynamic evaluation.

A reflex bladder contraction can be triggered by repeated suprapubic percussions. The technique is based on the increased reflex activity in the territory situated below the level of trauma, but not all the patients can obtain reliable contractions after percussion [**38**].

Conventional reeducation is not fulfilling all the aims of therapy, mainly because post voiding residual urine is always present due to the functional bladder outlet obstruction. The upper urinary tract remains at high risk, and urinary incontinence may also be present. Detrusor hypertrophy or lower urinary tract symptoms may still be present, so patients who use this voiding method require permanent attention, in order to avoid complications.

If this voiding option is associated with medical or surgical therapy, its results may be improved. Alpha-blockers give good results if used in high doses, but side effects, mainly arterial hypotension, are limiting its use [**39**]. Antispastic treatment is also effective in the treatment of detrusor-sphincter dyssinergia but its indications are also limited by side effects. The most effective therapy for detrusor-sphincter dyssinergia is the use of sedatives in high doses, but this cannot represent an option in the long run.

Endoscopic sphincterotomy dramatically improves voiding in more that 80% of cases, also improving the protection for both lower and upper urinary tracts. The main limitation of this technique is the urinary incontinence requiring permanent use of urine collecting devices.

**2. Intermittent catheterization**

Intermittent catheterization is the most effective voiding option for SCI patients, being the therapeutically gold standard. The method was first introduced by Guttmann in 1944, but it was not used because appropriate catheters were not available and also because the risk for urinary infections was considered to be high. The modern theory of *clean intermittent catheterization* was presented by Lapides in 1972. The two fundamental concepts are:

***1.** host resistance theory:* any bacteria introduced into the bladder are neutralized by a healthy tissue.

***2.** the frequency of catheterization is more important than sterility*.

Lapides underlines that self catheterization is not necessarily a sterile maneuver; if the patient cannot perform the catheterization by himself, the sterility is mandatory [**40**]. The success of this technique is dependent on a good indication and on careful training of the patient. Daily practice demonstrates an initial reluctance of the patient, but understanding the long term advantages and a good knowledge of the technique is usually enough to insure the success of self catheterization.

Both conventional reeducation and self catheterization do not solve the problem of high intravesical pressure during filling, so anticholinergic therapy should be associated in order to inhibit reflex bladder activity and to increase bladder capacity. If this treatment is not effective, a more invasive approach is to be used: detrusor paralysis using botulinic toxin (in specialized centers) or surgery for augmenting bladder volume.

**3. Other therapeutic options**

A relatively new option with promising results is electrical stimulation of the bladder; the technique aims to restore the physiological mechanisms of the lower urinary tract [**41**] by using intermittent electrical impulses on the anterior sacral nerve roots. The results published by vanKerrebroeck show the restoration of the normal activity of the lower urinary tract in 80% of cases, the main failure cause being device malfunction [**42**].

Indwelling urinary catheter is not a long term option for SCI patients not only because of the high risk for urinary tract infections but also because the catheter is limiting the mobility of the patient, being also a barrier before social reinsertion. A trial performed on a big number of patients and published in 2002 shows that the risk of bladder cancer is 71 times higher in patients with indwelling catheters [**43**]. Another trial published 13 years ago shows a 35% incidence of orchiepidydimitis in patients with indwelling catheter, compared to only 3% in patients performing intermittent catheterization [**44**]. The risk for upper urinary tract infections is 3 times higher in the group with indwelling catheterization. If none of the voiding options can be obtained after treatment or reeducation, a suprapubic catheter should be placed, taking into account its lower morbidity risk compared to indwelling catheterization. This option does not reduce the risk of bladder stones, so this aspect should be known in the follow up.

The follow up protocol should include clinical examination, ultrasonography and urodynamic evaluation. The patient should be examined twice a year in the first two years, once a year in the next five years and once every two years after that period.

A treatment is considered to be effective if it insures long term protection, a good quality of life and the social reinsertion of the patient. The success of the therapy is dependent on the compliance of the patient, and the compliance of the patient is usually dependent on the success of the therapy, so the relation between the patient and the physician is the key for a successful social reinsertion of the patient.
